# Supplementary material for: Objectively and subjectively measured physical activity levels in individuals with whiplash associated disorder and aged-matched healthy controls
Source: PLoS One. 2023 Oct 5;18(10):e0292629. doi: 10.1371/journal.pone.0292629 (PMC10553333; doi:10.1371/journal.pone.0292629)
Supplement: Attachment — Submitted filename: Response to reviewers.docx [file pone.0292629.s002.docx]

Thank you to the reviewer for the comments and feedback. Our response to each suggestion is detailed below.

1. In the Abstract, please add that the Bland-Altman analyses indicated an overestimation in MVPA and an underestimation in sedentary time by subjective methods.
   a. The following sentence was added to the abstract: *Bland-Altman analyses indicated that subjects over-reported MVPA and underestimated sedentary time using the IPAQ.*
2. In the Methods, please state where participants were recruited from.
   a. The following information was added to the methods: Participants were invited to attend two sessions *in Brisbane, Queensland, Australia*.
3. In the Methods, please state the specific cut-offs used for converting accelerometer counts to the different PA intensities (sedentary, light, moderate, vigorous, and MVPA) (line 141-142).
   a. The data have been added to the Methods section. …ActiGraph accelerometers *(sedentary ≤ 2860, light = 2861 - 4835, moderate = 4836 – 8452 cpm, vigorous = ≥ 8453)*.
4. Please add units to the x- and y-axis in Figure 2 – e.g., MVPA mpd
   a. ‘mpd’ has been added to axes in Figure 2
5. In the Results, please state the measurement tool (i.e., subjective) that is displaying an overestimation in MVPA (line 255-256) and an underestimation in sedentary time (line 263-264).
   a. ‘(IPAQ)’ has been added after the words over-reporting and under-reporting. …though Bland-Altman analysis highlighted the over-reporting *(IPAQ)* of MVPA by a mean of 17 mpd…; …Bland-Altman analysis highlighted the under-reporting *(IPAQ)* of sedentary time by a mean of 147 mpd…
